# Mutant p53 as a Therapeutic Target: The Report of Its Death Was an Exaggeration

**DOI:** 10.3390/ijms26136446

**Published:** 2025-07-04

**Authors:** Franck Toledo

**Affiliations:** 1 Hematopoietic and Leukemic Development Team, Centre de Recherche Saint-Antoine, 75012 Paris, France; franck.toledo@sorbonne-universite.fr; 2 INSERM UMRS938, 75012 Paris, France; 3 Sorbonne University, Saint-Antoine Campus, 75012 Paris, France

**Keywords:** p53, cancer, gain of function, inflammation

## Abstract

*TP53* is the most frequently mutated gene in human cancers. Many studies have reported oncogenic gain of function by mutant p53 and suggested that mutant p53 is a potential therapeutic target. In striking contrast, a recent approach relying on CRISPR-mediated mutagenesis led to the conclusion that mutant p53 removal in tumors had no therapeutic value. However, experimental limitations likely affected this study, including the difficulty of recapitulating the events leading to mutant p53 gain of function in cancer cell lines. Furthermore, a low statistical power may have masked the impact of mutant p53 removal in organoid-derived tumors. Independently, two studies focusing on the human hotspot mutant *TP53*^Y220C^ and its murine homolog *Trp53*^Y217C^ recently provided compelling evidence that mutant p53 can be a valid therapeutic target. Drugs designed to stabilize the mutant protein and restore wild-type p53 functions, or to inhibit the inflammatory effects associated with mutant p53, appear particularly promising.

## 1. Introduction

Missense mutations affecting the DNA binding domain of p53 are common in cancers [1]. Missense p53 mutants abrogate the canonical p53 tumor suppressive functions, but whether they may be more oncogenic than *TP53* loss has recently become extremely controversial.

The concept that cells expressing mutant p53 might have a selective advantage over cells lacking p53 was first proposed over 30 years ago [2]. Evidence in support of this came from expressing several p53 mutants in p53 null cells and finding increased plating efficiency in agar cell culture and faster tumor growth in nude mice [2]. Many reports in human cell lines and mouse models then confirmed this notion [3]. Furthermore, germline *TP53* mutations cause the Li–Fraumeni syndrome (LFS) of cancer predisposition [4], and the stratification of LFS patients according to *TP53* mutations indicated that, compared with nonsense mutations, missense p53 mutations may correlate with accelerated tumor onset or distinct cancer spectra [5]. Mouse models expressing some of the most frequent (“hotspot”) missense mutants indicated mechanisms that might explain how mutant p53 can accelerate tumorigenesis (although species-specific differences remain possible). In its wild-type (WT) form, p53 mainly acts as a homo-tetrameric transcription factor that, in response to various stresses, launches a large transcriptional program triggering cellular responses such as apoptosis, cell cycle arrest, senescence or metabolic changes [6]. Typically, p53 mutants lose the capacity to bind specific DNA sequences, leading to a loss of canonical WT p53 functions (LOF). Furthermore, in heterozygotes a p53 mutant may exert a dominant negative effect (DNE) by inhibiting the wild-type protein in hetero-tetramers [7]. Mutant p53 may also be stabilized in the cell and engage in aberrant interactions with other transcription factors, DNA helicase subunits or chromatin-modifying complexes, leading to the acquisition of oncogenic gain of function (GOF) properties [8,9,10]. Importantly, the finding of a mutant p53 GOF suggested that p53 mutants might be promising therapeutic targets [3].

However, the existence, or therapeutic relevance, of mutant p53 GOF has been contested in recent years. The analyses of a few p53 mutants in a mouse model of B cell lymphoma and in human acute myeloid leukemia suggested that they promoted tumorigenesis by DNE rather than GOF effects [11,12]. Furthermore, a recent study relying on CRISPR-based techniques to modify the *TP53* status of human cancer cell lines or cancer-derived organoids recently led to the conclusion that mutant p53 removal had no impact on the properties of tumor cells in vitro and in vivo [13]. The authors reached this conclusion after using CRISPR to remove mutant p53 in 16 cell lines from seven different cancer types and two colon-cancer-derived organoids, finding that it affected neither the proliferation and response to chemotherapeutic drugs of cells or organoids in culture, nor the growth and metastasis of tumor xenografts in mice [13]. The study, commendable for its scope and ambition, appeared so extensive and thorough that it cast some doubt about the reality, or at least the permanence and therapeutic relevance, of mutant p53 GOF [14,15].

## 2. Limitations of CRISPR-Mediated Mutagenesis of Cancer Cell Lines

Efforts to reconcile the contradictory data about mutant p53 GOF led to emphasis on the fact that the behavior of mutant p53 may vary depending on the cellular context [16], with researchers pointing to the technical limitations affecting CRISPR-based studies [17]. Lozano et al. proposed that multiple parameters, including tissue environment, immunological context, intracellular binding partners, mutant p53 protein conformation, stabilization and even time, are together required for mutant p53 GOF to arise, and that all these parameters are not easily recapitulated when using cancer cell lines [17]. Consistent with this interpretation, no GOF effects were initially observed after deep CRISPR-mediated *TP53* mutagenesis in the HCT116 cell line, but serial in vivo passaging of engineered HCT116 cells correlated with a stabilization of mutant p53 and increased cell migration and invasion [18]. Thus, as thoroughly discussed by Lozano et al. [17], when cancer cell lines are used to perform CRISPR-mediated mutagenesis, the observation of mutant p53 GOF may depend on experimental conditions.

## 3. CRISPR-Based Data from Organoid-Derived Tumors: Evidence of Insufficient Statistical Power

In their CRISPR-mediated mutagenesis experiments, Wang et al. performed mutant p53 removal not only in cell lines, but also in human colon-cancer-derived organoids, analyzed in culture or as xenograft tumors [13]. This is important because organoid-derived tumors are considered robust preclinical models [19]. The authors reported to have obtained similar results with tumor xenografts from cell lines and organoids, but this conclusion is debatable. Indeed, mutant p53 removal in cancer cell lines did not affect xenograft tumor growth in their experiments (see [13], Figure 6B,F). However, the removal of p53^R248W^ or p53^R175H^ in organoids correlated with a decrease in average tumor weight, although this was apparently statistically insignificant ([13], Figure 6I and Supplementary Figure S26B). Yet, the sample size was very low in these experiments: for each organoid line, only six xenograft tumors per group were analyzed [13]. By contrast, studies conducted by using genetically engineered mouse models compared much larger animal cohorts to provide robust evidence of mutant p53 GOF. For example, evidence of oncogenic GOF was recently obtained for *Trp53*^Y217C^, a mouse model of the human hotspot mutant *TP53*^Y220C^, by comparing the survival of 31 *Trp53*^Y217C/Y217C^ and 21 *Trp53*^−/−^ mice [20], whereas the comparison of only 6 mice per group would probably have been uninformative. It is thus reasonable to hypothesize that Wang et al. might have missed the effect of mutant p53 removal in organoids due to an insufficient statistical power in their study.

**Figure 1:**
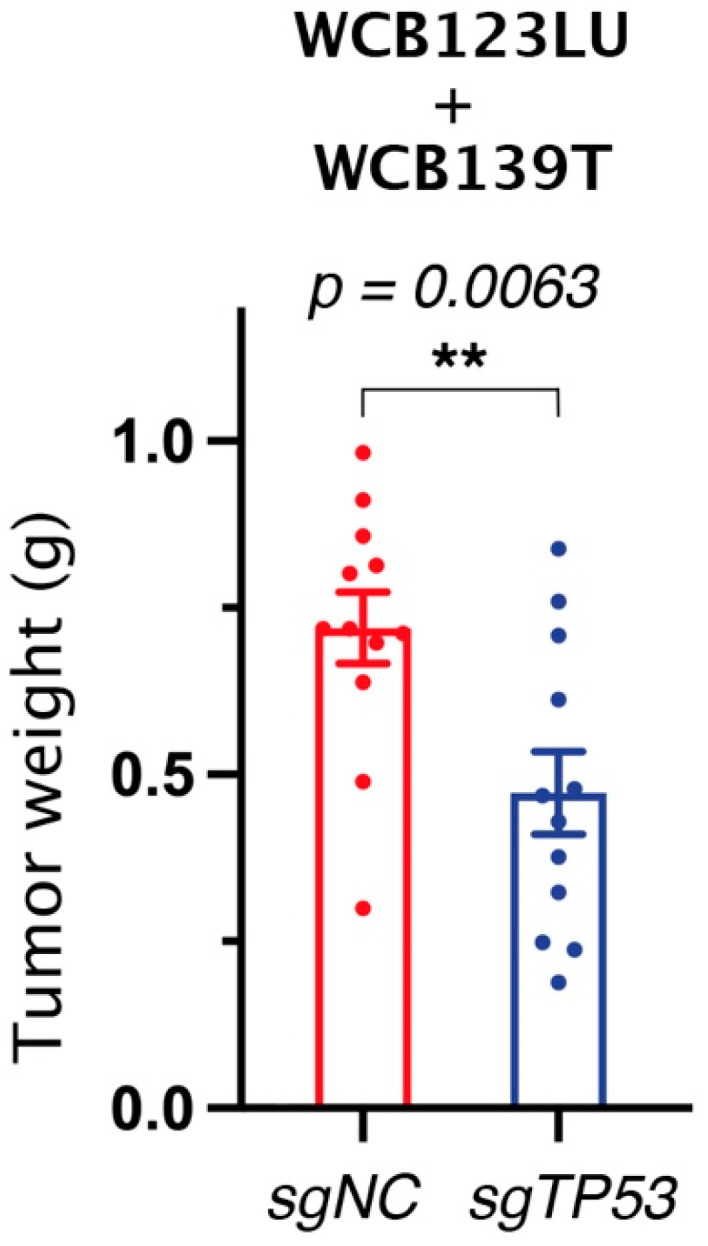
Evidence that the removal of mutant TP53 may impact the in vivo growth of human colon-cancer-derived organoids. Wang et al. [13] presented the growth in immune-deficient mice of human colon-cancer-derived organoids WCB123LU and WCB139T, which were either mutant *TP53*-expressing controls (referred to as *sgNC*) or their mutant *TP53*-deleted derivatives (*sgTP53*). They compared the weight of tumors at the ethical endpoint for 6 mice per organoid line: tumors for 6 WCB123LU *sgNC* and 6 WCB123LU *sgTP53* organoids are shown in Figure 6I, and tumors for 6 WCB139T *sgNC* and 6 WCB139T *sgTP53* organoids are shown in Supplementary Figure S26B, reporting similar experimental conditions [13]. Here, for the purpose of this commentary, both sets of data were re-plotted and analyzed together instead of separately in order to compare 12 *sgNC* with 12 *sgTP53* organoid tumors. Because experimental values were not available, each figure panel (Figure 6I or Figure S26B) was enlarged to A4 full-page format and the positions of individual points were measured. After re-plotting with Graphpad Prism 10.4.2, the resulting figure was superposed on the original panels by using layers in Adobe Photoshop in order to verify that individual points were accurately positioned in the graph. The data are presented as the mean ± SEM from 12 mice per group, analyzed with Graphpad Prism in a two-tailed unpaired Student’s *t* test (** = *p* < 0.01). By using a very small portion of the data from ref. [13] to generate a new figure for criticism/commentary with a research/scholarship purpose, this approach falls under the ‘fair use’ doctrine of copyright law.

To test this hypothesis, I gathered the organoid tumor data presented in ([13], Figure 6I and Supplementary Figure S26B), to evaluate the effects of mutant p53 removal in both organoid lines in the same figure. By comparing two groups of 12 tumors, this re-analysis indicated that mutant p53 removal in organoids may lead to a subtle but statistically significant decrease in tumor weight (Figure 1). This is consistent with a mutant p53 GOF whose impact was masked by a low statistical power in the initial analysis provided by the authors [13].

## 4. Recent Evidence That Mutant p53 Can Be a Valid Therapeutic Target

As discussed above, although Wang et al. provided extensive evidence that the deletion of mutant p53 had no effect in cancer cell lines [13], a re-analysis of their data obtained with organoid-derived tumors (Figure 1) may actually support the notion that targeting mutant p53 in patients’ tumors might be a valid therapeutic strategy. Importantly, two studies focusing on the human mutant *TP53*^Y220C^ or its murine homolog *Trp53*^Y217C^ recently provided compelling evidence that mutant p53 can be a relevant target.

*TP53*^Y220C^ is one of 12 highly recurrent *TP53* missense mutants, each representing at least 1% of the first *TP53* mutations compiled in the COSMIC mutation database [21]. This mutant was later found to exhibit a male-specific oncogenic GOF in gliomagenesis [22] and shown to be particularly frequent in myeloid neoplasms [23]. Unlike other hotspot mutants, it does not occur at a CpG site, so its occurrence does not result from the random deamination of an unstable 5-methyl cytosine [21]. Structural studies revealed that mutation from a tyrosine to a cysteine lowers the stability of the DNA binding domain, and that it creates a crevice that might be a target site for a mutant-selective stabilizing drug [24]. Rezatapopt (PC14586) is such a drug, specifically designed to reactivate the p53^Y220C^ mutant [25]. This molecule was recently reported to correct the mutant p53^Y220C^ protein by stabilizing the protein structure, hence restoring its DNA binding activity and its capacity to trigger a transcriptional program inhibiting tumor growth [25]. Its efficacy was shown in vivo in xenografts and syngeneic humanized mouse models, and in two patients [25]. Rezatapopt provides direct evidence that a mutant p53 can be a valid therapeutic target, and it is currently under evaluation in a phase II clinical trial. Of note, however, is the fact that it does not demonstrate the therapeutic relevance of targeting mutant p53 GOF per se because it acts by correcting a mutant p53 protein to restore wild-type p53 functions.

Independently, the analysis of *Trp53*^Y217C^, a mouse model of the *TP53*^Y220C^ human mutant, provided evidence that targeting mutant p53 GOF can be therapeutically relevant. A thorough analysis of this mouse model revealed that the *Trp53*^Y217C^ mutant exhibited a loss of canonical WT p53 functions (LOF), as well as GOF effects that could be oncogenic or teratogenic, whereas no significant DNE could be observed [20]. Importantly, inflammation was shown to underlie the GOF of several p53 mutants [10,26,27], and this was also true for the *Trp53*^Y217C^ mutant [20]. Compared to p53 loss, the *Trp53*^Y217C^ mutation caused accelerated tumor growth and increased metastasis in homozygous male mice, correlated with an increased expression of inflammation-related genes [20]. Consistent with other studies [10,28], the increased inflammation most likely resulted from the chromosomal instability caused by the mutant p53 [20]. Surprisingly, the *Trp53*^Y217C^ mutation also caused the perinatal death of most homozygous female mice, indicating a female-specific teratogenic GOF for this mutant [20]. Increased inflammation appeared as a likely explanation for the frequent female-specific perinatal death, because inflammation was proposed to promote neural tube defects or embryonic death in a few mouse models [29,30,31], and because male embryos might be protected by the anti-inflammatory properties of androgens [31,32,33]. Strikingly, the perinatal lethality of homozygous mutant females was partially rescued by administrating supformin in utero [20]. Supformin (LCC-12) is an inhibitor of the CD44–copper signaling pathway known to drive inflammation and cancer progression [34,35]. Thus, an anti-inflammatory drug could attenuate the GOF effects of a mutant p53 [20]. Because inflammation underlies the oncogenic GOF of other hotspot p53 mutants—such as *TP53*^R273H^ and *TP53*^G245D^ [10]—supformin appears as a potential drug to target the oncogenic GOF of many p53 mutants.

**Figure 2:**
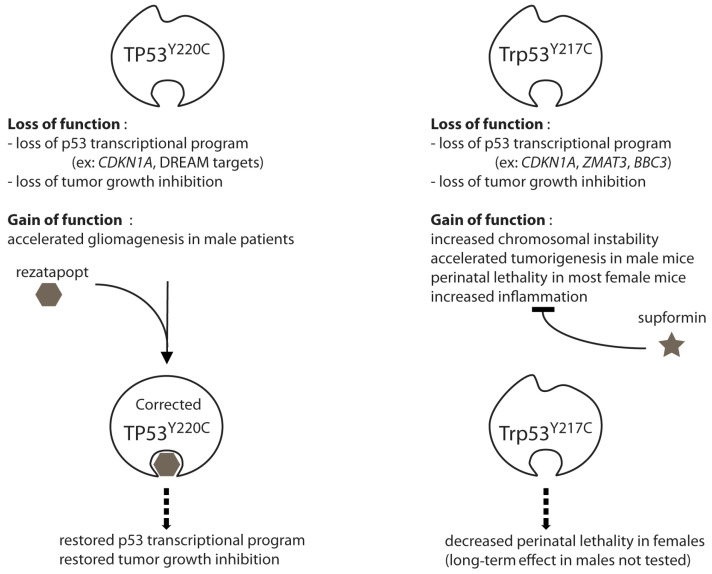
The evidence that mutant p53 is a valid therapeutic target. On the left, rezatapopt is a molecule that targets the TP53^Y220C^ mutant specifically. The molecule stabilizes the mutant p53 DNA binding domain, thereby restoring wild-type p53 functions. On the right, supformin does not target the Trp53^Y217C^ mutant per se. Rather, it inhibits inflammation, a GOF effect observed for this and several other p53 mutants. Both drugs were shown to have therapeutic effects in vivo [20,25]. For more details, see refs. [20,22,24,25,34].

In conclusion, whether directly through the structure-correcting rezatapopt or indirectly through the GOF-inhibiting supformin, the relevance of mutant p53 as a therapeutic target still lives on (Figure 2).

## Abbreviations

The following abbreviations are used in this manuscript:

CRISPR	Clustered regularly interspaced short palindromic repeats
DNE	Dominant negative effect
GOF	Gain of function
LFS	Li–Fraumeni syndrome
LOF	Loss of function
WT	Wild-type
